# National approaches to trichiasis surgical follow-up, outcome assessment and surgeon audit in trachoma-endemic countries in Africa

**DOI:** 10.1136/bjophthalmol-2019-315777

**Published:** 2020-07-26

**Authors:** Grace Mwangi, Paul Courtright, Anthony W Solomon

**Affiliations:** 1 Department of Surgery, Division of Ophthalmology, University of Cape Town, Cape Town, South Africa; 2 Kilimanjaro Centre for Community Ophthalmology, Division of Ophthalmology, University of Cape Town, Observatory, South Africa; 3 Department of Control of Neglected Tropical Diseases, World Health Organization, Geneva, Switzerland

## Abstract

**Background:**

Poor outcomes of trichiasis surgery, including postoperative trichiasis, are common in many trachoma-endemic countries in Africa. To improve outcomes, WHO recommends regular follow-up and outcome assessment of surgical cases plus audit of trichiasis surgeons.

**Aims:**

To assess national approaches to trichiasis surgical follow-up, outcome assessment and audit, and identify national targets for good surgical outcome (defined as the percentage of patients undergoing surgery for trichiasis remaining free of post-operative trichiasis for a defined interval after surgery).

**Methods:**

A cross-sectional survey was carried out between May and July 2018, involving all 29 known-trachoma-endemic countries in Africa. An emailed questionnaire was used to collect information on national targets for surgical outcomes, policies, monitoring and strategies to address underperformance by surgeons.

**Results:**

All national programmes provided information; 2 of the 29 had not yet implemented trichiasis surgery as part of their trachoma elimination programme. Findings from 27 countries are therefore reported. Only four countries reported having a national policy for trichiasis surgery follow-up and outcome assessment and only two had a national policy for conducting audits of trichiasis surgeons. Only 9 of the 27 countries had a cut-off point at which poorly performing surgeons would be instructed to discontinue surgery until retraining or other interventions had been undertaken.

**Discussion:**

To address the challenge of post-operative trichiasis and other poor outcomes, national trachoma programmes should create and implement policies and systems to follow up patients, assess surgical outcomes and monitor the performance of individual surgeons through post-surgical audits.

## Introduction

Preliminary analyses of population-based data from the Global Trachoma Mapping Project^1^ indicate that, varying by location, 10–75% of people who currently have trichiasis have already had trichiasis surgery on at least one affected eyelid.^2^ The factors driving the occurrence of post-operative trachomatous trichiasis (PTT) cannot be ascertained from cross-sectional survey data, but these data suggest that in some settings, a considerable proportion of trichiasis surgeries that need to be undertaken are revision procedures. Quality assurance procedures for trichiasis surgery should be reviewed to ensure that outcomes of primary procedures can be maximised.

WHO recommends regular follow-up of patients and programmatic monitoring of trichiasis surgery outcomes to help reduce the incidence of PTT and to facilitate its management where it is detected.^2^ WHO advises^3^ that all operated trichiasis patients be seen on the first post-operative day for eye patch removal and examination of the wound. Further follow-up at 8–14 days is recommended; this is essential if non-absorbable sutures are used.^2^ All patients should be followed up again 3–6 months after surgery.^2^ The primary purpose of each of these reviews is to allow the surgeon to assess the status of the operated eyelid(s) and make decisions, with the patient, regarding further management.

Parallel activities to improve outcomes of trichiasis surgery include supportive supervision and audit of trichiasis surgeons. The goal of an audit is to determine if individual surgeons have acceptable outcomes, need additional training and supervision or should discontinue providing surgery altogether. According to the Second Global Scientific Meeting on Trachomatous Trichiasis,^2^ for audit purposes, programmes should assess a sample of patients operated on by each individual practising surgeon. Data from an audit should be used to review the performance of new trichiasis surgeons 3–6 months after first certification, and of existing trichiasis surgeons as indicated.^2^ If a surgeon is found to have an unacceptably high incidence of PTT or other poor outcomes, retraining and more intensive supportive supervision by a more experienced surgeon is recommended.^4–6^

It is recognised that trichiasis patient follow-up, monitoring of outcomes and auditing of surgeons’ performance are challenging for trachoma programmes.^2^ Recommended policies and guidance on all aspects of trichiasis surgical service delivery have been developed by WHO and the International Coalition for Trachoma Control (ICTC), but adaption and adoption of these instruments at national level has been incomplete. There are structural and financial implications of implementation, which is made particularly difficult by the field environment: trachoma generally affects the poorest and most marginalised members of society, who often live in areas distant from health facilities.^7^ Such patients are often lost to follow-up after surgery, rendering post-operative care and monitoring of outcomes impossible.^8^

We undertook a survey to assess the availability of national policies and strategies for monitoring outcomes and for conducting surgical audits, and strategies for addressing poor surgical quality among surgeons in the 29 African countries where trachoma was still considered a public health problem in 2018.

## Materials and Methods

**Figure 1:**
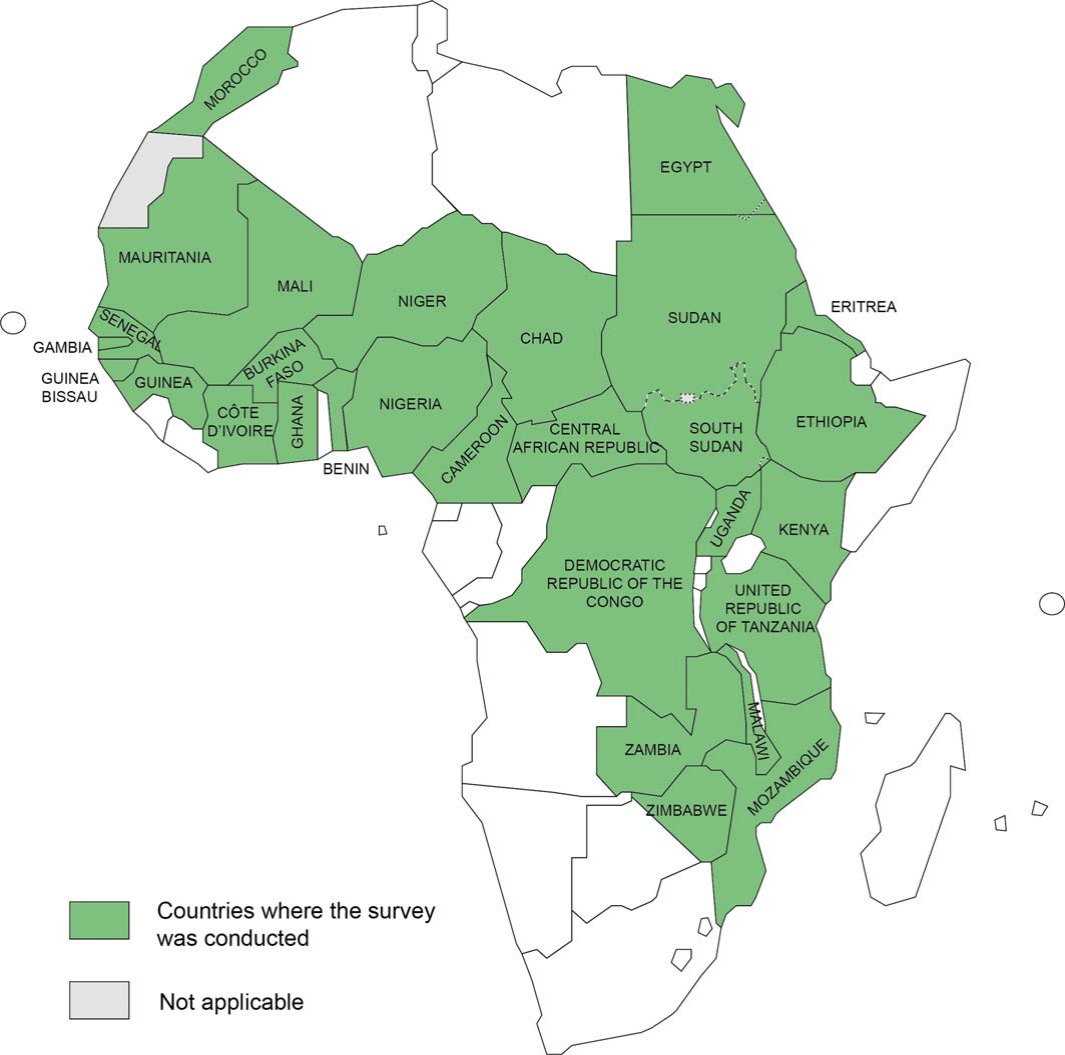
The 29 countries in Africa in which the survey was conducted. The boundaries and names shown and the designations used on this map do not imply the expression of any opinion whatsoever on the part of the World Health Organization concerning the legal status of any country, territory, city or area or of its authorities, or concerning the delimitation of its frontiers or boundaries. Dotted lines on maps represent approximate border lines for which there may not yet be full agreement. Credit: Illustrated by Alexei Mikhailov.

The survey was conducted in 29 trachoma-endemic countries in Africa (figure 1). Each national trachoma programme coordinator (or the equivalent) in these countries was invited to participate. Participants were recruited via email and requested to provide informed consent before completing a questionnaire. Ethical approval was obtained from the University of Cape Town Health Sciences Research Committee and the WHO Ethics Review Committee (0003034).

A single cross-sectional survey was carried out between April and July 2018. An emailed questionnaire (see online supplementary material) was used to collect information. Prior to formal use, the questionnaire was piloted to assess wording, content and format, with pilot respondents being one national trachoma programme coordinator and one ophthalmologist with extensive knowledge and experience in trachoma programmes. Feedback from this pretest was used to refine the questionnaire, which was then translated into French and Portuguese. Further validation through pilot testing was undertaken with intended respondents who were bilingual in either English and French or English and Portuguese, to ensure that the translated instruments were equivalent to the English original.

Survey responses were entered into Microsoft Excel, checked for completeness and verified against the original response forms. Responses in French and Portuguese were translated into English and checked (through back-translation) for correctness. Aggregated tables were generated in Excel. Qualitative data were analysed using thematic analysis and narrative synthesis.

## Results

All 29 eligible national coordinators consented and participated. Two of 29 trachoma-endemic countries had not yet commenced implementation of a trichiasis surgery programme; both were excluded from data analysis. Findings are presented for 27 countries.

**Figure 2:**
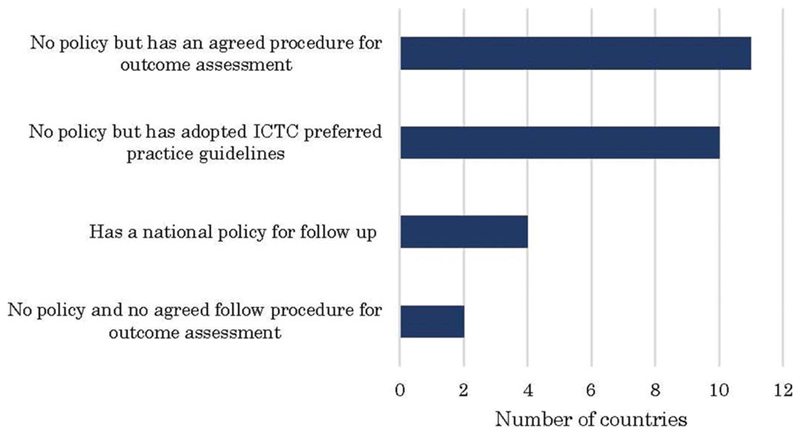
Availability of national policies for trichiasis surgery follow-up.

Only 4 (15%) of 27 countries reported having a national policy for follow-up of trichiasis patients after surgery (figure 2). Of the remaining 23, 11 did not have national policies or guidelines but reported that systems and structures for follow-up of patients after surgery were nevertheless in place; 10 countries had adopted the ICTC preferred practice guidelines^8–10^; and two countries had no policy or agreed on follow-up procedure for outcome assessment.

Sixteen (59%) of 27 countries reported mandating all three recommended follow-up points: 1 day after surgery, 1–2 weeks after surgery, and 3–6 months (or 1 year) after surgery. Of the remaining 11 countries, 4 countries mandated at least two follow-up points, 6 countries had only one mandated follow-up point, and 1 country did not have any mandated follow-up points.

Three countries had not set a national target for good surgical outcomes (defined as the percentage of patients undergoing trichiasis surgery remaining free of PTT at 3–6 months or 1 year). Among the remaining 24 countries, 3 reported having a target of 100% good outcomes, 14 countries had a target of 90%, 1 country had a target of 85%, and 6 countries had a target of 80%.

**Figure 3:**
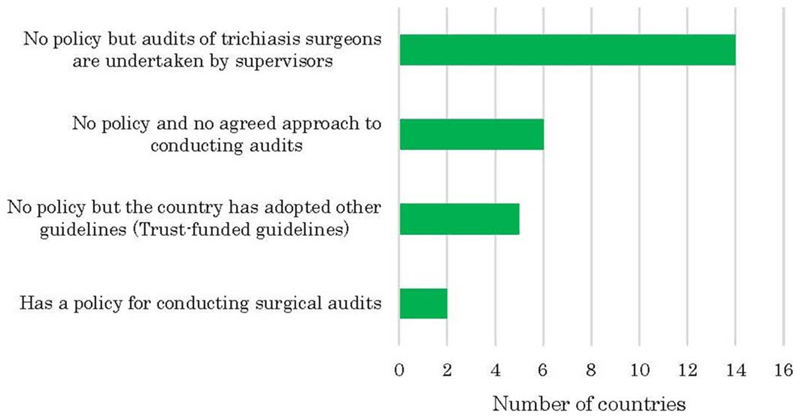
Countries with a national policy for conducting surgical audits.

Only two countries reported having a national policy regarding surgeon audits while 19 countries (70%) had no specific national surgeon audit policy but reported that audits of trichiasis surgeons were undertaken by supervisors (n=14) or had adopted other guidelines, such as the Queen Elizabeth Diamond Jubilee Trust funded surgical audit guidelines (n=5) (figure 3). These guidelines recommend the use of surgical audit as part of quality assurance to improve the quality of trichiasis surgeries delivered.^11^ The remaining six countries had neither a national policy nor an agreed approach to conducting audits of surgeons.

It is recommended that surgeons with poor outcomes (with the threshold for ‘poor’ being defined locally) should undergo a retraining and certification process.^11^ In this survey, only 10 (37%) of 27 countries reported having a nationally accepted threshold above which a surgeon should discontinue surgery. Variable thresholds were reported among those 10 countries; 30% poor outcomes (3 countries), 25% (1 country), 20% (4 countries), 15% (1 country) and 10% (1 country).

Of the 17 countries that reported having national targets for good outcomes (≤10% PTT), only 1 had a nationally defined threshold of 10% at which a surgeon should discontinue surgery until retraining or other interventions have been undertaken. Six countries reported that a surgeon-level incidence of 15–30% PTT would prompt this action, while 10 countries had no PTT threshold for surgeons to discontinue surgery.

Countries recommended different approaches to manage PTT. In 19 (70%) of 27 countries, cases of PTT or other complications were reportedly managed by another trichiasis surgeon who was more experienced than the original surgeon (n=13) or by a specially trained ophthalmologist (n=6). Seven countries reported that surgery for PTT was done by the same surgeon who had performed the first procedure. One country did not specify its approach for managing PTT.

Fifteen (88%) of the 17 countries that reported aiming for ≤10% PTT also reported that cases of PTT were either managed by a specially trained ophthalmologist or a more experienced trichiasis surgeon. The remaining two countries reported that post-operative cases were either managed by the surgeon who performed the first procedure or the surgeon who identified them during a surgical campaign.

Some key challenges highlighted by national coordinators included (1) lack of funds to support the full range of trichiasis-related activities; (2) limited national data to inform planning; (3) limited numbers of secondary eye care units for post-operative management; (4) lack of proper follow-up systems for trichiasis patients after surgery; (5) weak referral systems; (6) refusal by patients to undergo additional surgery; and (7) shortage of trained human resources to manage PTT.

## Discussion

In 2010, the third Global Scientific Meeting on Trachoma recommended that national programmes should routinely report outcomes of trichiasis surgery, with a target of achieving ≤10% PTT at 1 year after surgery.^12^ In November 2018, the fourth Global Scientific Meeting on Trachoma revised that recommendation, requesting that cases be divided into those that had minor vs major trichiasis (see below) pre-operatively, and suggesting that the timing of the final follow-up point be 3–6 months after surgery. For minor trichiasis (≤5 upper eyelid eyelashes touching the eyeball), the target was set as <10% PTT at the final follow-up point, while for major trichiasis (>5 upper eyelid eyelashes touching the eyeball), it was set as <20%.^13^

The work reported in this paper indicates that in 2018, prior to the change noted earlier, only half the trachoma-endemic countries in Africa had set national outcome targets consistent with the then-current (2010) WHO recommendation (ie, >90% good outcomes). We have recently reviewed the published literature and found that even in the often highly controlled confines of the research setting, a PTT incidence of <20% at 3–12 months after surgery is rarely achieved.^14^ Without the combined framework of policies and other health system elements to support the practice of follow-up and outcome assessment, however, not only is the incidence of poor outcomes likely to remain high, it will continue to be unrecorded and unremarked.

We therefore note with concern that in 2018, only 42% of countries in Africa reported having their own specific national policy for follow-up of trichiasis patients after surgery (15%) or having adopted the ICTC preferred practice guidelines^8^ (27%). Only 59% of countries expected surgeons to see patients at all three WHO-recommended follow-up points. Another 15% of countries promoted at least two follow-up periods: 1 day and 3–6 months (or 1 year) after surgery. Of the seven countries that did not have the second recommended follow-up (1–2 weeks), only four reported routinely supplying and using absorbable sutures which might have made that follow-up less important. The remaining three countries provide non-absorbable sutures which should be removed at 7–10 days. This may mean that many patients in these countries retain unremoved sutures long after surgery. Although studies have found that the use of absorbable sutures does not lower the risk of PTT,^15^ retained sutures can cause granulomata, promote infection and traumatise the cornea.^16^ Trachoma elimination programmes should consider using absorbable sutures in case patients miss the 7–10-day follow-up for suture removal.

Surgical audit provides a crucial link to ensure that policy is translated into individual practice and good outcomes. As an independent assessment of surgical outcomes achieved by a specific surgeon, audit is an important quality assurance tool for national programmes and should be part of ongoing supportive supervision. Unfortunately, our survey indicated that in 2018, audits were given inadequate attention in most trachoma-endemic countries in Africa. The lack of identification of a cut-off for determining which surgeons need retraining suggests that most countries were not using audits for decision-making. The implication is that surgeons with unacceptably high incidence rates of PTT might continue to operate. Even in countries that had programme-level targets for incidence of PTT, a minority had surgeon-level thresholds prompting action for discontinuation or retraining, suggesting a disconnect between the targets and the policies in place to help achieve them. It would be wrong, however, to focus exclusively on the capacity of audit to identify providers who are performing sub-optimally: supervision and audit with feedback improve general health worker performance, could encourage higher surgical productivity^17^ and might reduce the current excessive surgeon attrition rate.^18,19^

We acknowledge several limitations of our study. First, we relied on self-reports from national coordinators. Second, we collected information at national level in 29 countries, rather than on actual practice regarding the management of trichiasis and PTT. It is likely that the picture painted by national coordinators is an over-simplification of the true situation on the ground, where practices could well vary between different subnational administrative areas. National policies, however, are important drivers of local action. Third, our definition of ‘good surgical outcome’ was essentially limited to an absence of PTT. Other unfavourable outcomes of trichiasis surgery, such as granuloma or eyelid contour abnormality with or without eyelid closure defects may, unfortunately, also be common.^20^ It is possible (but by no means certain) that national policies and approaches for assessment of PTT will also help in monitoring for these other complications.

Availability of reliable, quality-assured and quality-controlled data on the prevalence of active trachoma and trichiasis^21,22^ have been key to an evidence-based expansion in work against trachoma in recent years.^23^ Surgical follow-up data are amenable to similar standardisation and quality-marking.^24^ The recent infusion of financial and technical support for trichiasis throughout Africa should, through concerted effort of governments and their partners, make the requisite improvements possible. Specific attention is needed now to ensure that programmes generate the appropriate data on trichiasis surgery outcomes and use them to drive better results for patients.

## Supplementary Material

Supplemental material is published online only. To view please visit the journal online (http://dx.doi.org/10.1136/bjophthalmol-2019-315777).

Supplementary material

## Data Availability

All data relevant to the study are included in the article or uploaded as supplementary information.
