# Supplementary material for: National approaches to trichiasis surgical follow-up, outcome assessment and surgeon audit in trachoma-endemic countries in Africa
Source: Br J Ophthalmol. 2020 Jul 27;105(7):904–8. doi: 10.1136/bjophthalmol-2019-315777 (PMC7611077; doi:10.1136/bjophthalmol-2019-315777)
Supplement: Supplementary data [file bjophthalmol-2019-315777supp001.pdf]

POST OPERATIVE TRICHIASIS IN AFRICA - ONLINE SURVEY QUESTIONNAIRE

Dear Participant,

You are invited to participate in this survey to assess the current national targets for good trichiasis surgical outcomes, national policies and strategies for monitoring outcomes and conducting surgical audits, and strategies for addressing poor surgical quality among surgeons in trachoma endemic countries in Africa. Your participation is voluntary.

The survey procedure involves filling the questionnaire provided below which will take approximately 30 minutes to complete. Please answer all questions as accurately as possible by ticking the appropriate box for each question or writing your response in blank space provided. All responses are confidential, and all data will be compiled without identification to the respondent or the country. Data collected during the survey will be stored electronically on a password-protected computer.

The survey findings will be used as a foundation to discuss additional strategies to reduce post-operative trichiasis. If the results of the survey are published, no names or identifiable characteristics will be used in the publication. By returning this form you are providing consent for the information to be included in the survey.

Kindly return the completed questionnaire to mwngra003@myuct.ac.za. If you have any questions about the study, please do not hesitate to contact: Professor Paul Courtright (pcourtright@kcco.net) or Dr Anthony Solomon (solomona@who.int).

Thank you in advance for your participation.

ELECTRONIC CONSENT

I have read and understood the nature and purpose of this study and I voluntarily agree to participate.

☐ Yes

☐ No

Full Name: Click or tap here to enter text.

Designation: Click or tap here to enter text.

Country: Select one.

Date: Click or tap to enter a date.

SURVEY QUESTIONS

Q.1. What is the national target for good trichiasis surgery outcome in your country? (that is, what is the % of trichiasis surgeries that your country seeks to have recorded as a good outcome [no post-operative trichiasis] at 3-6 months or the last routine time that follow up is scheduled?)

Click or tap here to enter text.

Q.2. Is there a national policy for trichiasis surgery follow up (time periods when you expect patients to return for assessment) and outcome assessment?

- ☐ Yes (*Please attach*)
- ☐ No policy (and no agreed follow up for outcome assessment)
- ☐ No specific policy but surgeons are expected to conduct follow up and assess outcome on their trichiasis cases
- ☐ No policy but the country has adopted ICTC preferred practice guidelines
- ☐ Other. *Please specify*. Click or tap here to enter text.

Q.3. How many weeks and months after trichiasis surgery is recommended (as national policy or otherwise) for a trichiasis patient to have a follow up examination? (*Please tick all the follow up periods*)

- ☐ 1-day post-operative
- ☐ 1-2 weeks
- ☐ 3-6 months
- ☐ 1-year post-operative
- ☐ Other. *Please specify*. Click or tap here to enter text.

Q.4 Is there a national policy for conducting an audit (an audit refers to an assessment of surgical outcomes of a surgeon undertaken by a supervisor) of trichiasis surgeons in your country?

- ☐ Yes (*Please attach*)
- ☐ No policy (and no agreed approach to conducting audits)
- ☐ No policy but the country has adopted other guidelines e.g. the Queen Elizabeth Diamond Jubilee Trust funded surgical audit guidelines (*Please attach)*
- ☐ No specific policy but audits of trichiasis surgeons are undertaken by supervisors
- ☐ Other. *Please specify*. Click or tap here to enter text.

Q.5. Is there a nationally accepted cut-off point (percentage of post-operative trichiasis by a surgeon) that is used to recommend that a surgeon should discontinue surgery until retraining or other interventions have been undertaken?

☐ Yes. *Please state the cut-off point in %* Click or tap here to enter text.

☐ No

Q.6. What are the current approaches for managing post-operative trichiasis in your country (please tick/list all approaches)

☐ Management by the surgeon who did the first procedure

☐ Management by another trichiasis surgeon who is more experienced

☐ Management by a specially trained ophthalmologist

☐ Other approaches. *Please specify*. Click or tap here to enter text.

Q.7 What challenges have you faced in managing post-operative trichiasis in your country? (*Please list all challenges*)

Click or tap here to enter text.

Q.8. What other strategies would you recommend for reducing post-operative trichiasis in your country? (*Please list all strategies*)

Click or tap here to enter text.

~END~

Thank you for taking part in the survey.
